# Reprogramming to a pluripotent state modifies mesenchymal stem cell resistance to oxidative stress

**DOI:** 10.1111/jcmm.12226

**Published:** 2014-02-14

**Authors:** Karina D Asensi, Rodrigo S Fortunato, Danúbia S dos Santos, Thaísa S Pacheco, Danielle F de Rezende, Deivid C Rodrigues, Fernanda C P Mesquita, Tais H Kasai-Brunswick, Antonio C Campos de Carvalho, Denise P Carvalho, Adriana B Carvalho, Regina C dos S Goldenberg

**Affiliations:** a Instituto de Biofísica Carlos Chagas Filho, Universidade Federal do Rio de JaneiroRio de Janeiro, Brazil; b Instituto Nacional de CardiologiaRio de Janeiro, Brazil

**Keywords:** induced pluripotent stem cells, mesenchymal stem cells, oxidative stress, reactive oxygen species, menstrual blood

## Abstract

Properties of induced pluripotent stem cells (iPSC) have been extensively studied since their first derivation in 2006. However, the modification in reactive oxygen species (ROS) production and detoxification caused by reprogramming still needs to be further elucidated. The objective of this study was to compare the response of iPSC generated from menstrual blood–derived mesenchymal stem cells (mb-iPSC), embryonic stem cells (H9) and adult menstrual blood–derived mesenchymal stem cells (mbMSC) to ROS exposure and investigate the effects of reprogramming on cellular oxidative stress (OS). mbMSC were extremely resistant to ROS exposure, however, mb-iPSC were 10-fold less resistant to H_2_O_2_, which was very similar to embryonic stem cell sensitivity. Extracellular production of ROS was also similar in mb-iPSC and H9 and almost threefold lower than in mbMSC. Furthermore, intracellular amounts of ROS were higher in mb-iPSC and H9 when compared with mbMSC. As the ability to metabolize ROS is related to antioxidant enzymes, we analysed enzyme activities in these cell types. Catalase and superoxide dismutase activities were reduced in mb-iPSC and H9 when compared with mbMSC. Finally, cell adhesion under OS conditions was impaired in mb-iPSC when compared with mbMSC, albeit similar to H9. Thus, reprogramming leads to profound modifications in extracellular ROS production accompanied by loss of the ability to handle OS.

## Introduction

Since their first derivation in 2006 by Takahashi and Yamanaka [1], the amount of data gathered on induced pluripotent stem cells (iPSC) has been astonishing [2]. However, iPSC oxidative metabolism has only begun to be explored. It is expected that these cells would recapitulate embryonic stem cell (ESC) behaviour in response to oxidative stress (OS). Nevertheless, iPSC properties are not identical to ESC, especially with regard to the epigenome [3]. These cells can retain methylation patterns from their originating cells, a phenomenon termed epigenetic memory, which has been shown to influence differentiation [4].

Most of the studies analysing OS in iPSC have been performed in the context of reprogramming efficiency. Oxidative stress leads to DNA damage and, in turn, senescence-related genes induced by DNA damage impair the reprogramming process [5]. Hence, strategies that reduce OS such as physiological oxygen tensions [6] and addition of antioxidants [7] lead to an increase in reprogramming efficiency.

In one of the few studies comparing iPSC to ESC, Armstrong *et al*. [8] show that reactive oxygen species (ROS) levels and mitochondrial mass are similar in these cell types, and significantly lower than in the human dermal fibroblasts from which iPSC were generated. In accordance to Armstrong*s data, Prigione *et al*. [9] also found a reduced mitochondrial content in pluripotent stem cells types. The loss of mitochondria seems to be a critical step of the reprogramming process, switching the somatic oxidative metabolism to a glycolytic-dependent state [10].

However, it remains to be defined how these cells respond to increases in OS and if the epigenetic memory can influence these responses. In this study, we studied iPSC generated from menstrual blood mesenchymal stem cells (mbMSC) [11]. Menstrual blood constitutes an unusual and interesting mesenchymal stem cell source as it is readily available and can be easily obtained [12,13]. In addition, a remarkable characteristic of these cells is that they survive the intense necrotic process suffered by the endometrium during the menstrual cycle, suggesting that they are resistant to OS. Therefore, the objective of this study was to investigate and compare OS responses in iPSC generated from mbMSC (referred to as mb-iPSC), ESC and adult mbMSC.

## Materials and methods

### Cell isolation and culture procedures

Menstrual blood was obtained from healthy women at the peak of flow. Cells were centrifuged and submitted to Histopaque gradient according to the manufacturer*s instructions. All experiments were performed in passage 5. Our local institutional review board approved this study and all donors provided signed informed consent.

Human ESC (H9 and HES3), human dermal fibroblast iPSC (ihFib3.2) and mb-iPSC were cultured under defined conditions with BraStem2 culture medium (LaNCE, Rio de Janeiro, Brazil) on BD hESC-qualified Matrigel™. Human mb-iPSC were generated in our laboratory from mbMSC as previously described [11]. ihFib3.2 was also generated in our laboratory as described in the Supporting Information.

All experiments using mbMSC were made with biological repeats, obtained from different donors. As the same is not possible for ESC and iPSC, as they are established cell lines, we performed independent experiments using different cultures to obtain replicates.

### Flow cytometry and cell differentiation

For flow cytometry, stainings were performed in a 100-μl volume per tube for 30 min. at 4°C using a 1:30 dilution. Samples were acquired in BD FACSAria IIu. The protocols used for osteogenic and adipogenic differentiation are described in the Supporting Information.

### Population doubling time

Cells were plated in gridded culture dishes. Each grid had a known area, allowing quantification. Random grids were counted daily starting after the cells were plated and henceforth until confluence was achieved.

### Karyotype analysis

mbMSC were maintained in culture with Colcemide for 2 hrs. Subsequently, they were dissociated, centrifuged and resuspended in KCl solution. Cells were centrifuged once more and resuspended in methanol and acetic acid. Cells were placed on slides and stained with Wright stain solution. At least 20 metaphases were analysed and the number of chromosomes was manually counted using LUCIA KARYO software.

### Reverse transcription and polymerase chain reaction

Total RNA was obtained with RNeasy mini kit (Qiagen, Valencia, CA, USA) and 1 μg was used for reverse transcription with High-Capacity cDNA Reverse Transcription kit (Applied Biosystems, New York, MA, USA). Quantitative real-time PCR was performed with Maxima SYBR Green qPCR Master Mix (Fermentas, Pittsburgh, PA, USA) on a 7500 Real-Time PCR System (Applied Biosystems). Data were analysed using the 2^−ddCt^ method. Oligonucleotide sequences are listed in Tables S1 and S2 (all from IDT).

### MTT assay

Cells were exposed to H_2_O_2_ during 2 hrs and maintained in culture for another 24 hrs with regular culture medium. Subsequently, MTT 0.5 mg/ml was applied. After 90 min., MTT was replaced by DMSO and absorbance was measured at 540 nm in Victor™ X4 microplate reader.

### Amplex red-HRP assay

Superoxide dismutase (SOD), HRP, Amplex Red, glucose were diluted in balanced salt solution and added to 10^5^ cells. Fluorescence was immediately measured with excitation and emission wavelengths of 530 and 595 nm, respectively, in Victor™ X4 microplate reader.

### DCF assay

Cells were dissociated and incubated with or without 100 μM of H_2_O_2_ for 15, 30 or 60 min. Subsequently, 10 μM of CM-H2DCFDA was added for 30 min. at 37°C. Mean fluorescence intensity was analysed by flow cytometry to determine intracellular ROS levels.

### Antioxidant enzyme activities

Briefly, cells were lysed and protein concentrations were determined by Bradford*s method. Catalase activity was measured by the disappearance of H_2_O_2_, forming water and oxygen, as previously described [14]. The total activity of SOD was determined according to the method described by Crapo *et al*. [15]. Glutathione peroxidase (GPx) activity was measured as previously described [16]. All of these methods are described in the Supporting Information.

### Cell adhesion assay

In the adhesion assay, cells were dissociated and 10^5^ cells were replated with different concentrations of H_2_O_2_. After incubation with H_2_O_2_, the medium was removed, and cells were washed and fixed with ethanol. After fixation, cells were stained with crystal violet. Absorbance was measured at 570 nm using Victor™X4 microplate reader.

### Statistical analyses

Data are presented as mean ± SD. All experiments were analysed using one-way or two-way anova with Bonferroni*s post-test, except PDT in which we used linear regression. All analyses were performed with GraphPad Prism 5.0 (La Jolla, CA, USA), and *P* < 0.05 was considered significant.

## Results

### Menstrual blood–derived cells have a mesenchymal phenotype

Mononuclear cells were isolated and easily expanded to at least passage 10 and acquired a fibroblast-like morphology as passages progressed (Fig. S1).

**Fig. 1:**
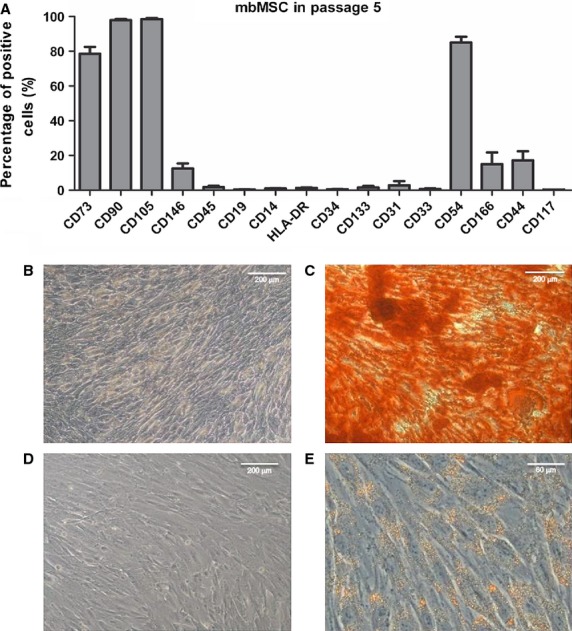
Flow cytometry and differentiation of mbMSC. (**A**) mbMSC (*n* = 11) presented a mesenchymal phenotype with a high percentage of cells positive for CD73, CD90 and CD105 and variable expression of adhesion molecules (CD146, CD54, CD166 and CD44). In addition, cells were predominantly negative for hematopoietic (CD45, CD19, CD14, HLA-DR, CD34 and CD117) and endothelial markers (CD133, CD31 and CD33). (**B** and **D**) Negative controls for osteogenic and adipogenic differentiations respectively. (**C**) Osteogenic differentiation of mbMSC (*n* = 3) showing calcium deposits in red. (**E**) Adipogenic differentiation of mbMSC (*n* = 3) showing lipid vacuoles in orange.

In passage 5, cells were predominantly positive for the human classic mesenchymal stem cell markers CD73, CD90 and CD105, while predominantly negative for hematopoietic (CD45, CD19, CD14, HLA-DR, CD34 and CD117) and endothelial (CD133, CD31, CD33) markers (Fig. 1A and Fig. S2). Adhesion molecules had a more heterogeneous expression with high levels of CD54 and variable amounts of CD146, CD166 and CD44 (Fig. 1A and Fig. S2).

Differentiation into osteogenic and adipogenic lineages was induced for 21 days. Figure 1B and D show cells that were maintained in regular culture medium. Osteogenic differentiation promoted the formation of calcium deposits in the extracellular matrix, as shown in red (Fig. 1C), whereas adipogenic differentiation promoted the accumulation of cytoplasmic lipid vacuoles, as shown in orange (Fig. 1E). These data fulfil the criteria defined by the International Society for Cellular Therapy [17] for mesenchymal stem cells.

**Fig. 2:**
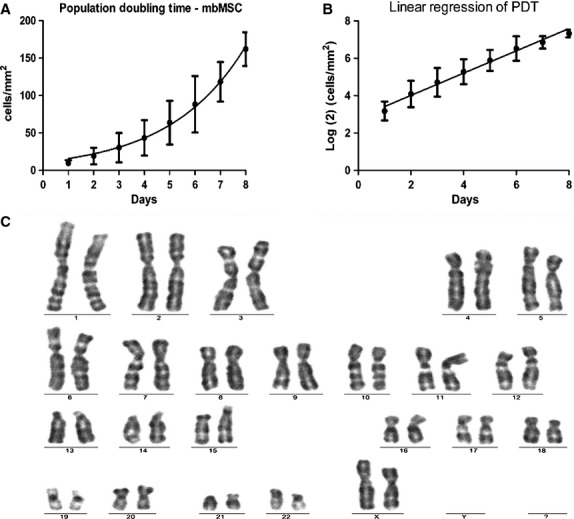
Population doubling time and karyotype of mbMSC. Passage 5 mbMSC (*n* = 8) exhibited exponential growth (**A**), and population doubling time was derived from the linear regression (**B**). (**C**) Representative image of mbMSC karyotype in passage 5 (*n* = 3).

Population doubling time (PDT) was 37.4 ± 4.08 hrs in passage 5, demonstrating the rapid growth rate of mbMSC. Exponential growth curves and linear regression are shown in Figure 2A and B. Colony forming unit assay showed formation of 7.8 ± 3.1 colonies for every 10^5^ plated cells. Chromosomal stability of mbMSC was also investigated because of its importance for large-scale expansion of these cells. G-banding analysis from three independent samples showed that mbMSC maintained diploid cells without chromosomal abnormalities, such as translocation or segregation, and none of these alterations was found in passage 5 (Fig. 2C) or 10 (data not shown).

### Pluripotent stem cell characterization

Embryonic stem cell (H9 and HES3) and iPSC (mb-iPSC and ihFib3.2) exhibited rounded-shape morphology and high nucleus-to-cytoplasm ratio (Fig. S3). All cultures expressed OCT4 and NANOG as shown in Figure S4, demonstrating the maintenance of pluripotency along the passages.

### Reprogramming modifies production and susceptibility to reactive oxygen species

**Fig. 3:**
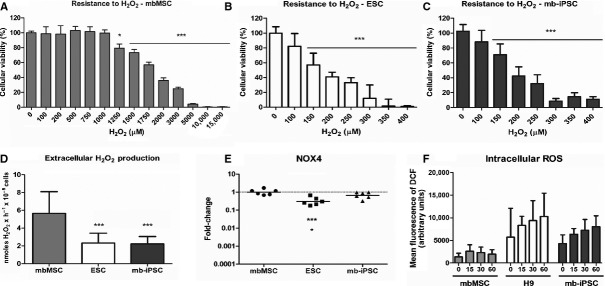
Production and susceptibility to reactive oxygen species. MTT assay showing cell viability in response to increasing doses of H_2_O_2_ in mbMSC (*n* = 6; **A**), H9 (*n* = 4; **B**) and mb-iPSC (*n* = 4; **C**). (**D**) Extracellular production of H_2_O_2_ by Amplex Red-HRP assay showing a significantly lower production in H9 (*n* = 6) and mb-iPSC (*n* = 6) when compared with mbMSC (*n* = 6; ***P < 0.001). (**E**) NOX4 expression was significantly lower in ESC when compared with mbMSC (***P < 0.001) and mb-iPSC (*P < 0.05). (**F**) Intracellular production of reactive oxygen species (ROS) by DCF assay. 0, 15, 30 and 60 indicate the duration of the exposure to H_2_O_2_ in minutes before the addition of DCF. There is a clear tendency to an increase in intracellular ROS amounts as exposure increases in pluripotent stem cells, although this is not significant. On the other hand, two-way anova revealed a significant difference in overall ROS levels among the three cell types analysed.

Given that mbMSC impressively survive the necrosis process that occurs during endometrial tissue shedding, we investigated their susceptibility to ROS. Cell viability was evaluated by MTT assay in response to crescent doses of H_2_O_2_. The H_2_O_2_ dose necessary to decrease cell viability by 50% (IC_50_) was 1812 ± 148 μM and cell viability only started to diminish after the dose of 1250 μM in mbMSC (Fig. 3A). Comparatively, mb-iPSC had an IC_50_ of 180 ± 26 μM and viability was already reduced at 100 μM of H_2_O_2_ (Fig. 3C). This behaviour was quite similar to the one observed for H9, which had an IC_50_ of 190 ± 42 μM (Fig. 3B). In addition, IC_50_ for ihFib3.2 and HES3 were 83 ± 14 and 86 ± 11 μM respectively (Fig. S5A and B).

In addition, extracellular production of H_2_O_2_ was almost threefold lower in mb-iPSC when compared with mbMSC, and similar to H9 production (Fig. 3D). As NADPH oxidases (NOX) are important contributors to ROS production, we investigated their expression by quantitative RT-PCR. These enzymes are transmembrane proteins that transport electrons across biological membranes to reduce oxygen to superoxide and hydrogen peroxide. NOX2 was expressed in all cell types, with no differences between them, while we did not detect the expression of NOX1 or NOX3 in any of the samples (data not shown). NOX5 was expressed in mbMSC, while pluripotent stem cells showed no expression of this transcript (data not shown). Furthermore, H9 had a significantly lower expression of NOX4 when compared with mbMSC and mb-iPSC (Fig. 3E).

Intracellular ROS production was also studied in mbMSC, mb-iPSC and H9 using DCF assay. The addition of H_2_O_2_ did not change the amount of intracellular ROS when comparing different time-points (15, 30 and 60 min.) to baseline (zero) within the same cell type, even though there is a clear tendency to an increase in pluripotent stem cells. However, when considering intracellular ROS amounts in different cell types, there is a significant difference among mbMSC, H9 and mb-iPSC (Fig. 3F). Importantly, there were no differences in autofluorescence in the fluorescein isothiocyanate channel when unstained cells were analysed (data not shown). This indicates that intracellular ROS concentrations tend to increase in pluripotent stem cells upon exposure to H_2_O_2_ when compared with mbMSC, which could be caused by a reduced expression of antioxidant enzymes.

### mb-iPSC have lower expression and activity of antioxidant enzymes

**Fig. 4:**
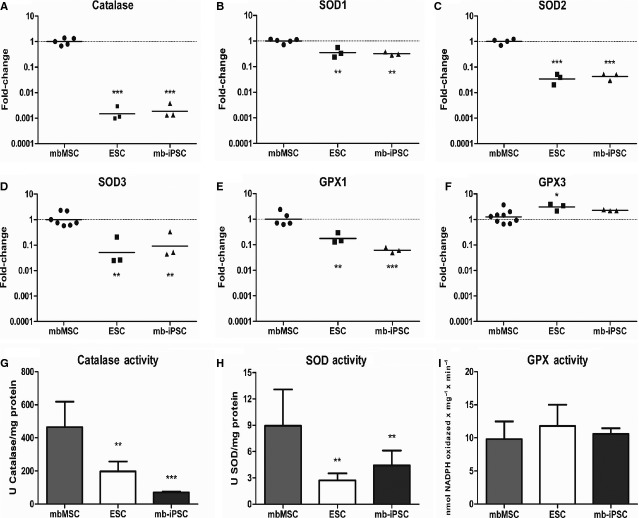
Antioxidant enzyme expression and activity. (**A**–**F**) Quantitative RT-PCR data of antioxidant enzymes. Data are expressed as fold-change in relation to mbMSC and plotted in a logarithmic scale. All enzymes, except for GPx 3, were significantly downregulated in pluripotent stem cells. (**G** and **H**) Antioxidant enzyme activities showing a significant reduction in catalase and SOD in pluripotent stem cells (*P < 0.05, **P < 0.01 and ***P < 0.001 compared with mbMSC).

The expression of several antioxidant enzymes was measured by quantitative RT-PCR. mb-iPSC and H9 had 590- and 749-fold lower expression of catalase when compared with mbMSC respectively (Fig. 4A). Superoxide dismutase 2 and 3 mRNAs were also lower in mb-iPSC and H9 compared with mbMSC, in a range of 14- to 31-fold reduction (Fig. 4C and D). Superoxide dismutase 1 reduction was more discrete and very similar in mb-iPSC and H9 (Fig. 4B), while GPx 1 was 16-fold lower in mb-iPSC and sixfold lower in H9 (Fig. 4E). Curiously, GPx 3 was approximately three- and twofold higher in H9 and mb-iPSC respectively (Fig. 4F).

As a reduction at the mRNA level does not necessarily indicate that the enzyme is less active, we performed assays to measure the activity of antioxidant enzymes. In accordance to quantitative RT-PCR results, catalase activity was reduced by 6.64- and 2.36-fold in mb-iPSC and H9 respectively (Fig. 4G). Superoxide dismutase activity was also reduced in mb-iPSC (2.04-fold) and H9 (3.33-fold; Fig. 4H). In addition, SOD activity was also significantly lower in HES3 and ihFib3.2 when compared with mbMSC (Fig. S5C), while there were no differences between pluripotent stem cells. Finally, consistent with the discrete differences found at the mRNA level, GPx activity was similar in mbMSC, mb-iPSC and H9 (Fig. 4I).

### Oxidative stress impairs adhesion of pluripotent stem cells

**Fig. 5:**
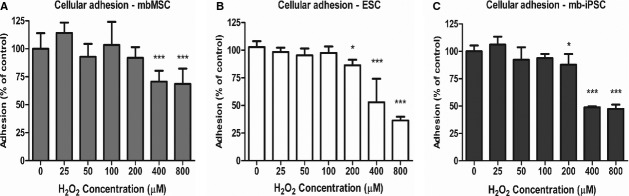
Cell adhesion under oxidative stress. Cells were dissociated and replated under increasing concentrations of H_2_O_2_. There was a significant reduction in adhesion in (**B**) H9 (*n* = 6) and (**C**) mb-iPSC (*n* = 6) at 200 μM of H_2_O_2_ (*P < 0.05 compared with control), while (**A**) mbMSC (*n* = 6) adhesion remained similar to control levels. At 400 and 800 μM of H_2_O_2_, all cell types presented reductions in adhesion (***P < 0.001 compared with control); however, the percentage of reduction was more pronounced in pluripotent stem cells.

Finally, we decided to test if the higher susceptibility of pluripotent stem cells to ROS would result in an impaired adhesion capability compared with mbMSC. Cells were plated with different concentrations of H_2_O_2_ and adhesion was estimated after 2 hrs. At 200 μM of H_2_O_2_, adhesion of H9 and mb-iPSC was significantly impaired, while mbMSC maintained adhesion in similar levels to control. Above 400 μM, adhesion was compromised in all cell types, even though the percentages of reduction were more pronounced in pluripotent stem cells (Fig. 5).

## Discussion

After the excitement promoted by the generation of induced pluripotent stem cells in 2006, researchers have dedicated enormous efforts to better understand the reprogramming process and the biological characteristics of these cells. In this context, interest in pluripotent stem cell physiology has increased. In fact, two reviews on this subject have recently been published [18,19]. To support their high proliferation rates, pluripotent stem cells are predominantly anabolic and tend to inactivate catabolic pathways. Accordingly, energy production is achieved through glycolysis rather than oxidative phosphorylation, which is dependent on the reduction in mitochondrial content [18,19].

Prigione *et al*. [9] have shown that ESC and iPSC have a lower mitochondrial DNA copy number when compared with human foreskin fibroblasts, as well as a lower mitochondrial mass. In addition, the mitochondria of pluripotent stem cells had an immature morphology, which led them to conclude that pluripotent stem cells are in a ‘low oxidative stress state’. Our work provides data showing that extracellular production of ROS is also lower in pluripotent stem cells when compared with adult mbMSC, giving further support to the ‘low oxidative stress state’ concept. Armstrong *et al*. [8] found similar results when comparing pluripotent stem cells to human dermal fibroblasts. Mitochondrial mass, number and superoxide levels were reduced in undifferentiated ESC and iPSC when compared with adult fibroblasts. Interestingly, they show that even though pluripotent stem cells had similar levels of total intracellular ROS production, mitochondrial production was lower in iPSC, suggesting an alternative source of ROS in these cells [8].

Reactive oxygen species, such as anion superoxide and hydrogen peroxide, can be formed as a by-product of different reactions, but only the family of enzymes called NADPH oxidases produces ROS as their main function. This family has seven members (NOX1 to NOX5, DUOX1 and DUOX2) that present different tissue distributions, subcellular localization and expression levels. Although H9 showed a significantly reduced expression of NOX4 when compared with mbMSC and mb-iPSC, this did not result in a lower extracellular H_2_O_2_ production when compared only with mb-iPSC. Therefore, this could not explain the differences between mbMSC and pluripotent stem cells. The higher extracellular H_2_O_2_ generation found in mbMSC can be attributed to NOX5 expression, as this enzyme is located at the plasma membrane and its mRNA was expressed in mbMSC, while undetectable in H9 and mb-iPSC. Moreover, intracellular ROS levels tended to increase in pluripotent stem cells upon exposure to H_2_O_2_, while mbMSC presented discrete variations in ROS levels. Finally, the overall effect of exposure to H_2_O_2_ was significantly different between mbMSC and pluripotent stem cells, suggesting that mbMSC might have more efficient antioxidant defences.

The ‘low oxidative stress state’ suggested by Prigione *et al*. is in agreement with the concept that ESCs require mechanisms to prevent and repair damage that might accumulate over successive generations. As ROS are major promoters of cell damage, it would be expected that pluripotent stem cells would possess highly efficient OS defence mechanisms. In fact, it has been reported that both murine [20] and human [21] ESCs present a proficient antioxidant defence, which tends to be reduced as differentiation progresses. Nevertheless, although it has been demonstrated that cell reprogramming requires a transition from an oxidative to glycolytic state [10], it is difficult to predict what would be the effect of reprogramming on OS defences. One would expect, from the data available on ESC, that reprogramming would increase antioxidant enzyme function. Our data show quite the contrary. Even though H9 and mb-iPSC had similar responses in all of our experiments, they were extremely sensitive to OS. Their viability was much lower in response to increasing concentrations of hydrogen peroxide; cell adhesion was diminished and addition of this substance to their culture media led to significant increases in intracellular ROS levels when compared with mbMSC. This indicated that antioxidant defences were more efficient in mbMSC and prompted us to study antioxidant enzyme expression and activities. As expected, the reductions observed in catalase and SOD expression and activities of mb-iPSC were quite striking in comparison with mbMSC. These data show that, at least for menstrual blood–derived mesenchymal cells, reprogramming diminished antioxidant defences. Given the published data showing that differentiation tends to reduce antioxidant enzyme expression [8,21] and increase sensitivity to ROS [22], and as reprogramming is considered a reversion of differentiation, this constitutes an apparent contradiction. Collectively, these findings show that the modulation of antioxidant defences upon reprogramming or differentiation is dependent not only on the process itself but also on the cell type. Thus, one cell type may suffer a reduction in their antioxidant defences upon reprogramming and, if differentiated to an unrelated cell type, present further reductions in these defences.

In conclusion, reprogramming leads to remarkable changes in cell morphology, differentiation capacity, growth properties, epigenome, energetics and redox homoeostasis. Our study demonstrates that the reduction in oxidative metabolism is accompanied by decrease in antioxidant defences in menstrual blood–derived induced pluripotent stem cells, indicating an important mechanism by which these cells could maintain low levels of ROS production coupled with a high sensitivity to changes in OS.

## Supporting information

**Figure S1** Light microscopy of mbMSC in culture. Cells were adherent to plastic and presented a spindle-shaped morphology in passages 5 (A) and 10 (B).

**Figure S2** Flow cytometry histograms showing cell surface phenotype of mbMSC. Isotype controls are shown in black and primary antibody stained cells are shown in blue. The percentage of positive events is indicated on the upper right corner.

**Figure S3** Light microscopy of ESC (H9: A and B; HES3: E and F) and iPSC in culture (mb-iPSC: C and D; ihFib3.2: G and H). ESC and iPSC grew as colonies and presented a rounded-shape with high nucleus/cytoplasm ratio (scale bar: 200 μm in A, C, E and G; 60 μm in B, D, F and H).

**Figure S4** Expression of pluripotency genes in ESC and iPSC by immunofluorescence. H9, HES3, mb-iPSC and ihFib3.2 presented nuclear expression of core transcription factors OCT4 and NANOG, shown in red. In blue, nuclei were stained with either DAPI or TO-PRO 3 (scale bar: A–L 10 μm; M–X 50 μm).

**Figure S5** Cell viability in response to increasing doses of H_2_O_2_. MTT assay shows a significant reduction in cell viability with doses above 60 μM and 75 μM in HES3 (A; IC_50_ 86 ± 11 μM) and ihFib3.2 (B; IC_50_ 83 ± 14 μM) respectively (****P* < 0.0001). (C) SOD activity was lower in pluripotent stem cells when compared with mbMSC (**P* < 0.05, ***P* < 0.01). No differences were found among H9, mb-iPSC, HES3 and ihFib3.2.

**Table S1** List of primers for antioxidant enzymes used in qRT-PCR experiments. **Table S2** List of primers for NADPH oxidases used in qRT-PCR experiments.
