# Supplementary figures and images for: Reprogramming to a pluripotent state modifies mesenchymal stem cell resistance to oxidative stress
Source: J Cell Mol Med. 2014 Feb 14;18(5):824–31. doi: 10.1111/jcmm.12226 (PMC4119388; doi:10.1111/jcmm.12226)

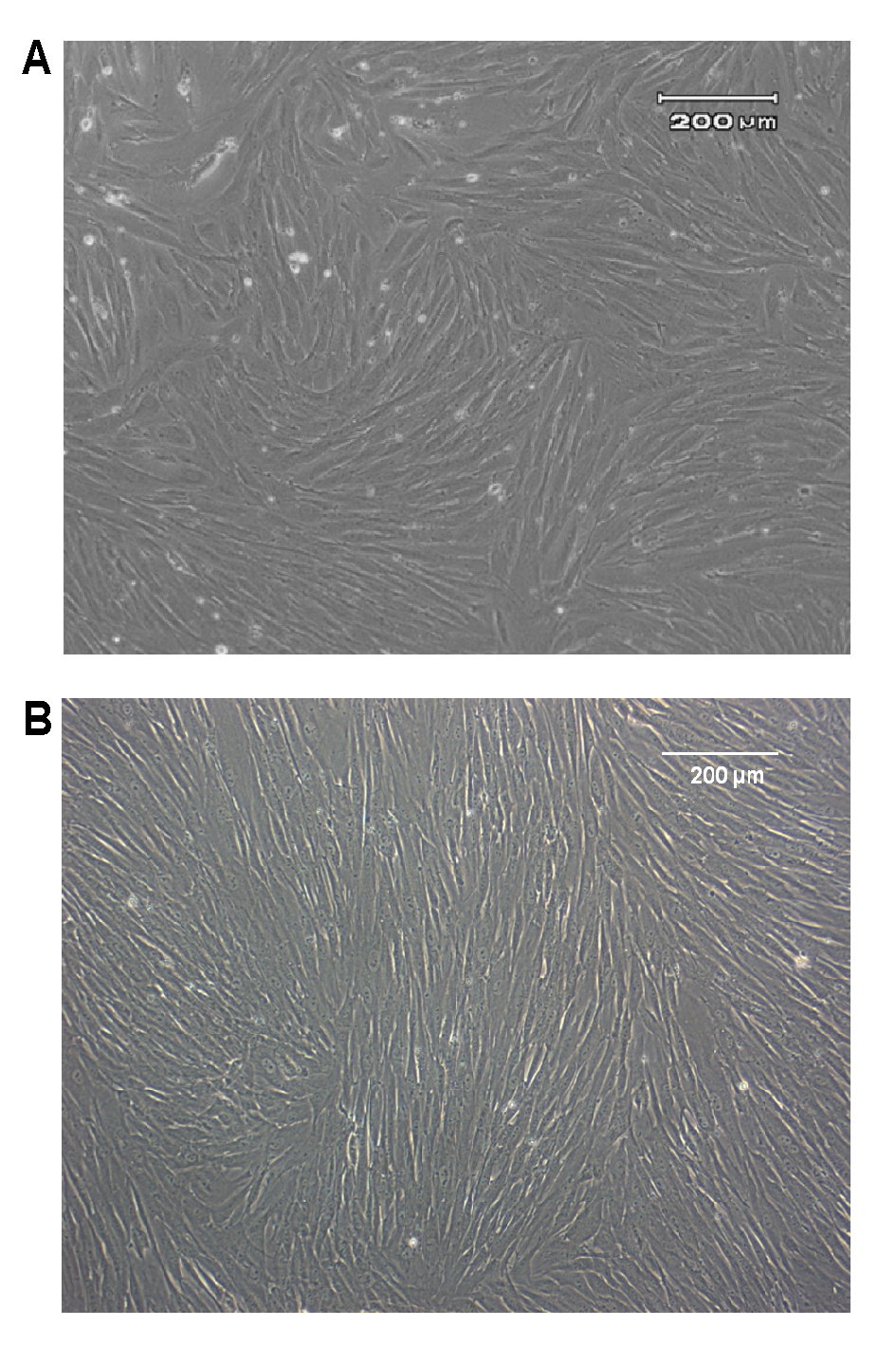

Supplement: Supplementary file 1 — Figure S1 Light microscopy of mbMSC in culture. Cells were adherent to plastic and presented a spindle-shaped morphology in passages 5 (A) and 10 (B). [file jcmm0018-0824-SD1.tif]

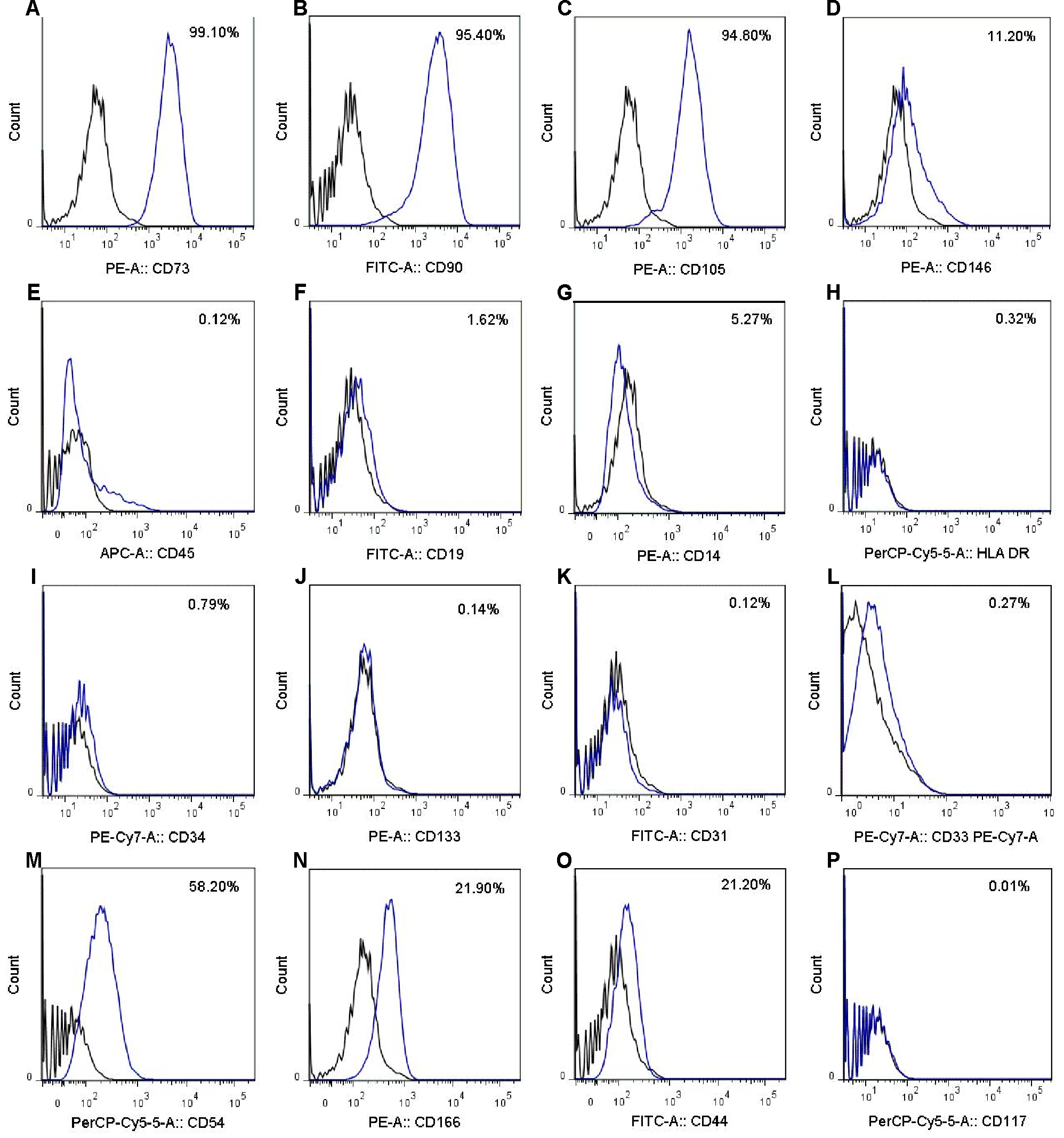

Supplement: Supplementary file 2 — Figure S2 Flow cytometry histograms showing cell surface phenotype of mbMSC. Isotype controls are shown in black and primary antibody stained cells are shown in blue. The percentage of positive events is indicated on the upper right corner. [file jcmm0018-0824-SD2.tif]

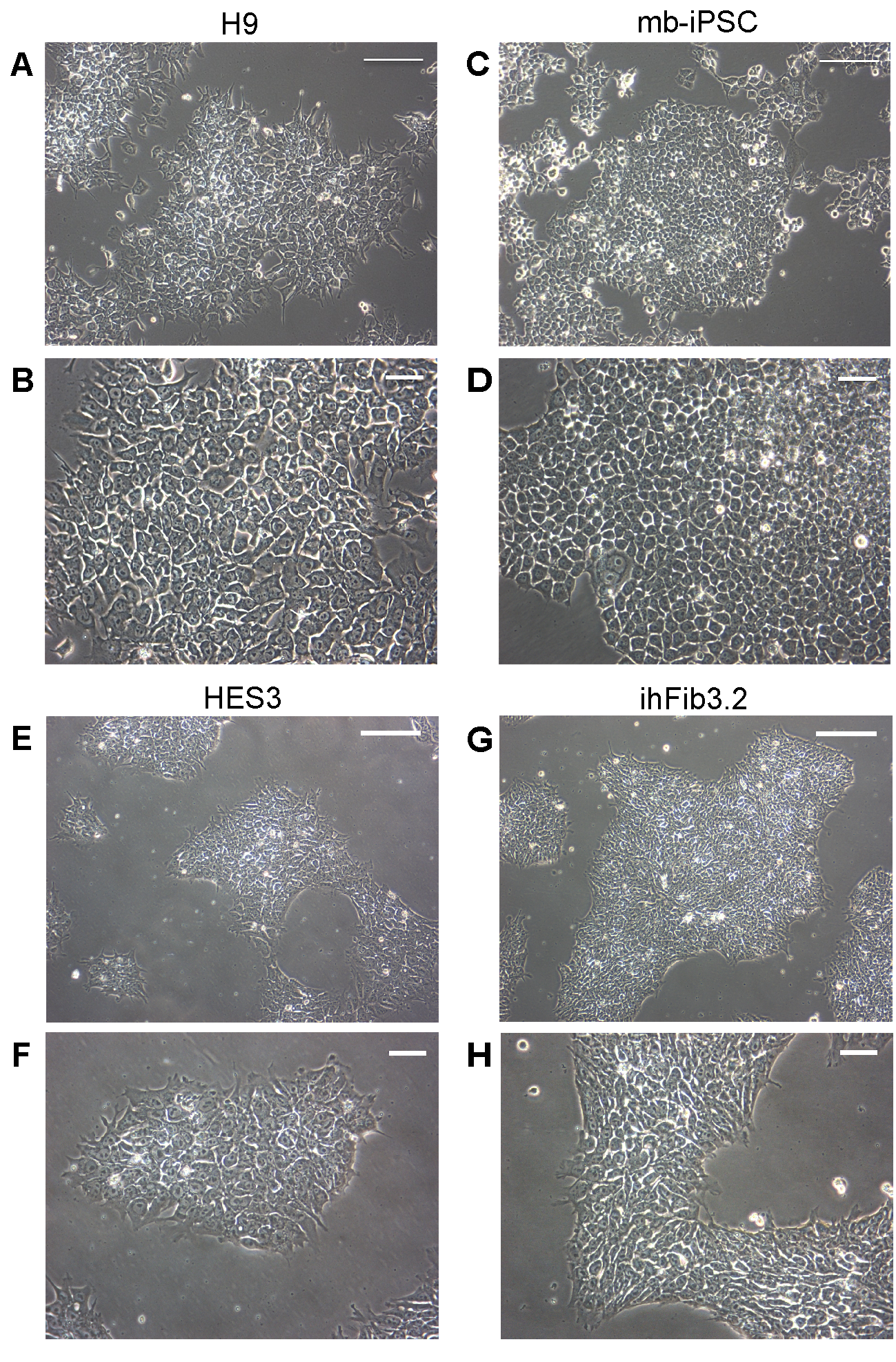

Supplement: Supplementary file 3 — Figure S3 Light microscopy of ESC (H9: A and B; HES3: E and F) and iPSC in culture (mb-iPSC: C and D; ihFib3.2: G and H). ESC and iPSC grew as colonies and presented a rounded-shape with high nucleus/cytoplasm ratio (scale bar: 200 μm in A, C, E and G; 60 μm in B, D, F and H). [file jcmm0018-0824-SD3.tif]

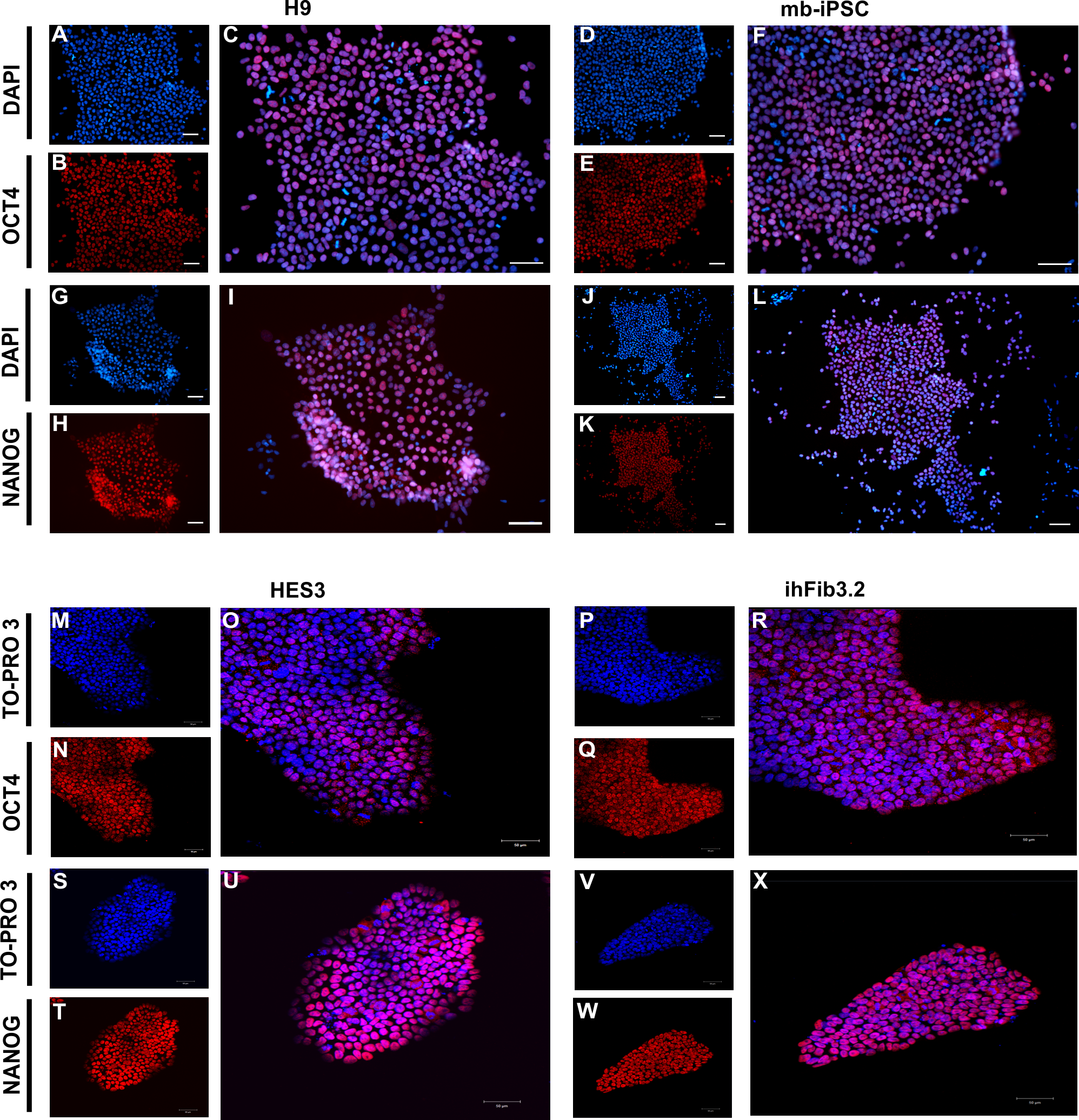

Supplement: Supplementary file 4 — Figure S4 Expression of pluripotency genes in ESC and iPSC by immunofluorescence. H9, HES3, mb-iPSC and ihFib3.2 presented nuclear expression of core transcription factors OCT4 and NANOG, shown in red. In blue, nuclei were stained with either DAPI or TO-PRO 3 (scale bar: A–L 10 μm; M–X 50 μm). [file jcmm0018-0824-SD4.tif]

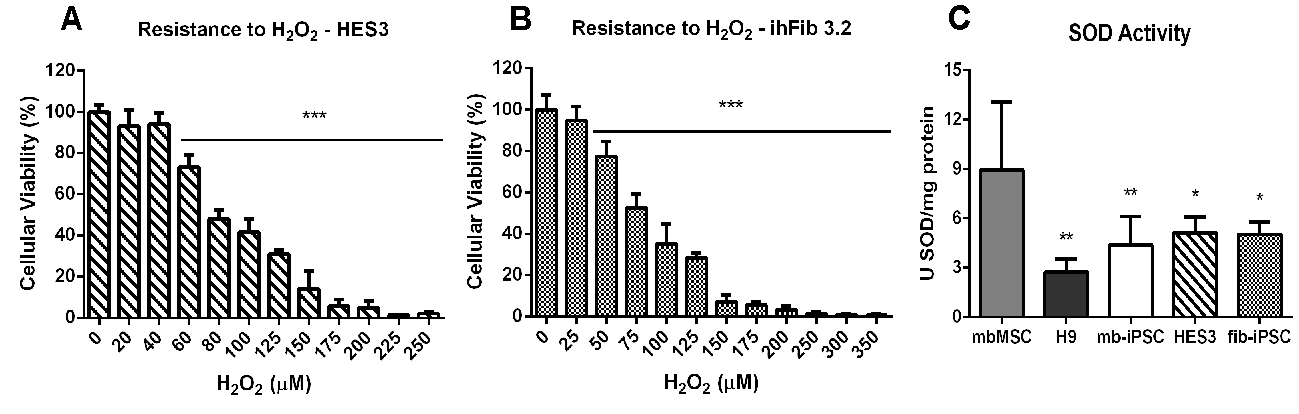

Supplement: Supplementary file 5 — Figure S5 Cell viability in response to increasing doses of H2O2. MTT assay shows a significant reduction in cell viability with doses above 60 μM and 75 μM in HES3 (A; IC50 86 ± 11 μM) and ihFib3.2 (B; IC50 83 ± 14 μM) respectively (***P < 0.0001). (C) SOD activity was lower in pluripotent stem cells when compared with mbMSC (*P < 0.05, **P < 0.01). No differences were found among H9, mb-iPSC, HES3 and ihFib3.2. [file jcmm0018-0824-SD5.tif]
