# Supplementary material for: Reprogramming to a pluripotent state modifies mesenchymal stem cell resistance to oxidative stress
Source: J Cell Mol Med. 2014 Feb 14;18(5):824–31. doi: 10.1111/jcmm.12226 (PMC4119388; doi:10.1111/jcmm.12226)
Supplement: Supplementary file 6 [file jcmm0018-0824-SD6.docx]

**SUPPORTING INFORMATION**

**1.** **SUPPLEMENT MATERIALS AND METHODS:**

**1.1. Cell Isolation and Culture Procedures**

Menstrual blood was obtained from healthy women (n=18) between 18 and 26 years of age. All donors were under regular gynecological care and had a normal cervical smear exam. Blood was collected at the peak of flow in a sterile recipient containing 5 mL of phosphate-buffered saline (PBS) supplemented with 100 U/mL of penicillin, 100 mg/mL of streptomycin and 0.5 mM of EDTA. Cells were centrifuged and submitted to Histopaque gradient (Histopaque 1.077 g/mL, Sigma-Aldrich) according to the manufacturer’s instructions. Mononuclear cells were collected, washed in PBS and cultured in DMEM-high glucose supplemented with 20% fetal bovine serum (FBS, Gibco), 2 mM of L-glutamine and penicillin-streptomycin. Cells were expanded and experiments were performed in passage 5. Our local institutional review board approved this study and all donors provided signed informed consent.

Human ESC (H9 and HES3), human dermal fibroblast iPSC (ihFib3.2) and mb-iPSC were cultured under defined conditions with BraStem2 culture medium (LaNCE, Rio de Janeiro, Brazil) on BD hESC-qualified Matrigel^TM^ (BD Biosciences). Media was changed every 24 hours and cells were passaged using enzymatic dissociation with TrypLE^TM^ or collagenase IV (both from Invitrogen). Human mb-iPSC were generated in our laboratory from mbMSC as previously described [[11](#_ENREF_14)]. ihFib3.2 was generated using lentiviral vectors, which were produced using a five plasmid transfection system (pHAGE-EF1α-STEMCAA, VSV-G, Tat, Rev and Gag/Pol) in HEK293FT packaging cells (Invitrogen) with FuGENE6 transfection reagent (Roche). These plasmids were kindly donated by Dr. Gustavo Mostoslavsky [23]. Viral particles were concentrated by ultracentrifugation (45,000 x g, 4°C, 90 min) and frozen at -80°C. Approximately 100,000 fibroblasts were seeded on 35 mm plastic culture plates and transduced with concentrated virus (MOI 1) in the presence of Polybrene Infection/Transfection Reagent® (8 μg/mL) (Millipore). Cells were harvested after 7 days and seeded on BD Matrigel hESC-qualified matrix (BD Biosciences) with mTeSR1 culture medium (StemCell Technologies). iPS clones were picked 20 – 30 days after transduction based on morphology and cells were expanded and characterized.

**1.2. Flow Cytometry and Cell Differentiation**

For flow cytometry, cells were dissociated with trypsin-EDTA, washed once and ressuspended in PBS 0.5% BSA. Stainings were done in a 100 µL volume per tube for 30 minutes at 4°C using a 1:30 dilution. All antibodies were purchased from BD Biosciences, except for CD133 (Miltenyi Biotec). Samples were acquired in BD FACSAria IIu and analyzed in FlowJo 9.4.10.

For osteogenic differentiation, cells were submitted to a 21-day culture protocol with DMEM supplemented with 10% FBS, penicillin-streptomycin, 10^-7^ M of dexamethasone, 10 mM of β-glycerophosphate and 0.5 µM of ascorbic acid. After this period, cells were fixed and stained with Alizarin Red 1%. Adipogenic differentiation protocol was identical, except for β-glycerophosphate and ascorbic acid that were substituted for 0.45 mM of isobutyl-methylxanthine and 2.07 µM of human insulin. Staining was performed with Oil Red O 0.2%.

**1.3. Population Doubling Time**

Cells were plated in 35 mm gridded culture dishes (Nunc) (2x10^4^ cells/dish). Each grid had a known area of 4 mm^2^, allowing quantification of the number of cells per mm^2^. Four random grids were counted daily starting 24 hours after the cells were plated and henceforth until confluence was achieved. An exponential growth curve was built and transformed by plotting the y-axis in logarithm to base 2. Linear regression was performed and PDT was calculated from the reciprocal of the slope of the linear function obtained.

**1.4. Karyotype Analysis**

mbMSC were maintained in culture with 1.2 mM of Colcemide (Sigma-Aldrich) for 2 hours. Subsequently, they were dissociated by enzymatic digestion and centrifuged at 300 x g for 8 minutes. The precipitate was resuspended in preheated 75 mM KCl solution (Vetec) and incubated for 20 minutes at 37°C. Cells were centrifuged once more and resuspended in methanol and acetic acid (3:1) (Merck). This procedure was repeated three times and cells were placed on slides. The slides were incubated at 90°C for 1 hour. Finally, cells were stained with Wright stain solution (Sigma-Aldrich) for 4 minutes. At least 20 metaphases were analyzed and the number of chromosomes was manually counted using LUCIA KARYO software.

**1.5. Reverse Transcription and Polymerase Chain Reaction**

Total RNA was obtained with RNeasy mini kit (QIAGEN) and 1 µg was used for reverse transcription with High-Capacity cDNA Reverse Transcription kit (Applied Biosystems). Quantitative real-time PCR was performed with Maxima SYBR Green qPCR Master Mix (Fermentas) on a 7500 Real-Time PCR System (Applied Biosystems). All experiments were done in duplicates and GAPDH was used as housekeeping gene. Data were analyzed using the 2^-ddCt^ method. Samples which had Ct values greater than 35 were considered undetectable. Oligonucleotide sequences are listed in Table S1 and S2 (all from IDT).

**1.6. MTT Assay**

Susceptibility to death of different cell types to crescent doses of hydrogen peroxide was studied using MTT assay. Cells were exposed to H_2_O_2_, diluted in DMEM high-glucose with 20% FBS in the case of mbMSC or BraStem2 in the case of pluripotent stem cells, during 2 hours. After this period, the media was changed to DMEM 20% FBS or BraStem2 without H_2_O_2_ and cells were maintained in culture for another 24 hours. Subsequently, MTT 0.5 mg/mL was applied in the dark at 37°C. After 90 minutes, MTT was replaced by DMSO, which was incubated for 5 minutes, and absorbance was immediately measured at 540 nm in Victor^TM^ X4 microplate reader (PerkinElmer).

**1.7. Amplex Red-HRP Assay**

Superoxide dismutase (200 U/mL, Sigma-Aldrich), HRP (1 U/mL, Roche Life Sciences), Amplex Red (100 µM, Molecular Probes), glucose (2 mg/mL, Gibco) were diluted in balanced salt solution and added to 10^5^ cells. Fluorescence was immediately measured with excitation and emission wavelengths of 530 and 595 nm respectively in Victor^TM^ X4 microplate reader. A standard curve was performed with known concentrations of H_2_O_2_. The results were expressed as nmol H_2_O_2_ x h^-1^ x 10^5^ cells.

**1.8. DCF Assay**

Cells were dissociated with trypsin-EDTA and incubated at 37°C with or without 100 µM of H_2_O_2_ for 15, 30 or 60 minutes in DMEM high-glucose 0.2% FBS without phenol red. Incubation was performed using the same number of cells in all samples (2x10^5^). Subsequently, 10 µM of CM-H2DCFDA (DCF, Invitrogen) were added for 30 minutes at 37°C. Samples were placed on ice and mean fluorescence intensity was acquired by flow cytometry (BD FACSAriaIIu) to determine intracellular ROS levels. Dead cells were excluded using DAPI staining. Unstained cells were used to determine basal fluorescence levels in the FITC channel. Data were analyzed using FlowJo 9.4.10.

**1.9. Antioxidant Enzyme Activities**

Briefly, cells were lysed in a buffer composed of 50 mM Tris-HCl (pH 7.6), containing 5 mM EDTA, 0.01 mM dithiothreitol (DTT), 0.01 mg/mL aprotinin and 0.2 mM phenylmethylsulfonyl fluoride (PMSF). Protein concentrations were determined by Bradford’s method.

Catalase activity was measured by the disappearance of H_2_O_2_, forming water and oxygen, as previously described [[19](#_ENREF_19)]. A control experiment was done to assess the decrease of light absorption of the reaction medium (50 mM potassium phosphate buffer pH 7.0, Triton X-100 0.002%, 0.1 mM EDTA, 15 mM hydrogen peroxide, in a final volume of 1 mL) at 240 nm for about 2 minutes. Subsequently, the cell homogenate was added and light absorption continued to be monitored for another 2 minutes. Catalase activity was obtained by the difference between the rate of decrease in absorbance with and without the cell homogenate. The activity was calculated from the amount of H_2_O_2_ consumed per minute, using the molar extinction coefficient of H_2_O_2_ (43.6 M^-1^.cm^-1^). Enzyme activity was expressed in units (micromoles of consumed H_2_O_2_ per minute) per milligram of protein.

The total activity of SOD was determined according to the method described by Crapo and colleagues (1977). In this method, the anion superoxide (O^2-^) generated from xanthine-xanthine oxidase system reduces cytochrome c, causing an increase in light absorption at 550 nm. The addition of homogenate containing SOD causes dismutation of H_2_O_2_, preventing the reduction of cytochrome c. The reaction medium was composed of 50 mM potassium phosphate buffer (pH 8.0) containing 0.1 mM EDTA, 0.01 mM potassium cyanide, 0.02 mM cytochrome c and 0.05 mM xanthine, in a final volume of 1 mL. The reaction was triggered by adding xanthine oxidase (8 mU/mL) to the reaction medium and the reduction of cytochrome c was evaluated by the increasing absorption of light at 550 nm for about 1 minute and 30 seconds. Then, 100 μg of cell homogenate were added and the reduction of cytochrome c continued to be measured for 2 minutes. SOD activity was obtained by the difference in cytochrome c reduction with and without the cell homogenate. A 50% decrease in the rate of cytochrome c reduction was considered a SOD unit. The total SOD activity was expressed as units per milligram of protein.

GPx activity was measured as previously described [[21](#_ENREF_21)]. The reaction medium was composed of 100 mM potassium phosphate buffer, 1 mM EDTA, 0.15 mM NADPH, 0.5 mM reduced glutathione, 240 mU/mL glutathione reductase and 1.2 mM tert-butyl hydroperoxide. Baseline absorbance of 960 µL of the reaction medium was measured at 340 nm for 5 minutes. Cell lysate (40 µL) was added and absorbance was measured for another 5 minutes. GPx activity was calculated subtracting NADPH oxidation with or without the addition of cell lysate.

**1.10. Cell Adhesion Assay**

In the adhesion assay, cells were dissociated, counted and 10^5^ cells were replated with different concentrations of H_2_O_2_ (0, 25, 50, 100, 200, 400 and 800 µM) in triplicates. After two hours of incubation at 37°C with H_2_O_2_, the medium was removed, cells were washed three times with PBS and fixed with ethanol. After fixation, cells were stained with 0.05% (w/v) crystal violet and methanol was used for dilution. Aliquots of 150 μL were transferred to a 96-well plate and the absorbance was measured at 570 nm (A570) using Victor^TM^X4 microplate reader. Cell adhesion corresponded to the ratio between A570 in the presence and absence of H_2_O_2_ multiplied by 100.

**1.11. Immunofluorescence**

For immunofluorescence, cells were plated in coverslips coated with Matrigel and fixed with paraformaldehyde 4%. After 3 washes with PBS, cells were blocked and permeabilized using PBS with 0.5% of bovine serum albumin (BSA) and 0.3% Triton X-100 for 30 minutes at room temperature. Immunofluorescence was performed using OCT4A and NANOG primary antibodies (1:200, Cell Signaling Technology) diluted in PBS with 0.5% BSA overnight at 4°C. The secondary antibody was Cy3-AffiniPure Donkey Anti Rabbit IgG (1:1000; Jackson Research) diluted in PBS with 0.5% BSA for 1 hours at room temperature. Nuclei were stained with DAPI or TO-PRO®-3 Iodide (Molecular Probes). Fluorescence images were recorded using a digital camera attached to Carl Zeiss Apotome microscope or Zeiss LSM 510 Meta confocal microscope.

1.12. CFU Assay

After Histopaque centrifugation of menstrual blood cells, mononuclear cells were counted and plated at differents concentrations in 96-well plates. Concentrations of cells per well used were: 5x10^5^, 10^5^, 5x10^4^, 10^4^, 5x10^3^, 10^3^, 5x10^2^. The medium was composed of DMEM high-glucose supplemented with 20% FBS and 10^-6^M of hydrocortisone. After plating, cells were maintained in CO_2_ incubator for one week without manipulation. Subsequently, after the first week, 50% of medium was changed and cells were cultured for 7 more days. At the end of the protocol, cell were stained with Giemsa, positive wells were counted and frequency was analysed using L-Calc Stem Cell software.

Supplementary Table S1: List of primers for antioxidant enzymes used in qRT-PCR experiments

| **Gene** | **Sense primer sequence**  **Antisense primer sequence** | **Size** |
| --- | --- | --- |
| **CAT** | TGG ACA TCG CCA CAT GAA TG  GCC GCA TCT TCA ACA GAA AG | 132 bp |
| **SOD 1** | GTC CTC ACT TTA ATC CTC TAT CCA G  AGT CAC ATT GCC CAA GTC TC | 83 bp |
| **SOD 2** | GTA GCA CCA GCA CTA GCA G  CGT TGA TGT GAG GTT CCA GG | 150 bp |
| **SOD 3** | TGA ACT GGC CCA ATG ACT G  TCC TGA AAA GAG AGC TGC AC | 133 bp |
| **GPX 1** | GAC TAC ACC CAG ATG AAC GAG  TCG AAG AGC ATG AAG TTG GG | 176 bp |
| **GPX 3** | CTG CTT TCC CTG CTC CTG  GCT CCG TAC TCG TAA ATG GTG | 107 bp |
| **GAPDH** | ACC ATG GGG AAG GTG AAG GT  CAT GGG TGG AAT CAT ATT GG | 163 bp |

Supplementary Table S2: List of primers for NADPH oxidases used in qRT-PCR experiments

| **Gene** | **Sense primer sequence**  **Antisense primer sequence** | **Size** |
| --- | --- | --- |
| **NOX 1** | CCT GAG TCT TGG AAG TGG ATC  ACG CTT GTT CAT CTG CAA TTC | 147 bp |
| **NOX 2** | AGG AGT TTC AAG ATG CGT GG  TTG AGA ATG GAT GCG AAG GG | 142 bp |
| **NOX 3** | ACT GGG ACG AAA ATA CTG ACG  GAG AGC TTT AGG TCC ACA GAA G | 146 bp |
| **NOX 4** | TCA CAG AAG GTT CCA AGC AG  ACT GAG AAG TTG AGG GCA TTC | 140 bp |
| **NOX 5** | TGT TCA TCT GCT CCA GTT CC  ACA AGA TTC CAG GCA CCA G | 147 bp |
| **GAPDH** | ACC ATG GGG AAG GTG AAG GT  CAT GGG TGG AAT CAT ATT GG | 163 bp |

**Suplemmentary Figure Legends**

Supplementary Figure 1: Light microscopy of mbMSC in culture. Cells were adherent to plastic and presented a spindle-shaped morphology in passages 5 (A) and 10 (B).

Supplementary Figure 2: Flow cytometry histograms showing cell surface phenotype of mbMSC. Isotype controls are shown in black and primary antibody stained cells are shown in blue. The percentage of positive events is indicated on the upper right corner.

Supplementary Figure 3: Light microscopy of ESC (H9: A and B; HES3: E and F) and iPSC in culture (mb-iPSC: C and D; ihFib3.2: G and H). ESC and iPSC grew as colonies and presented a rounded-shape with high nucleus/cytoplasm ratio (Scale bar: 200 μm in A, C, E and G; 60 μm in B, D, F and H).

Supplementary Figure 4: Expression of pluripotency genes in ESC and iPSC by immunofluorescence. H9, HES3, mb-iPSC and ihFib3.2 presented nuclear expression of core transcription factors OCT4 and NANOG, shown in red. In blue, nuclei were stained with either DAPI or TO-PRO 3 (Scale bar: A-L 10 μm; M-X 50 μm).

Supplementary Figure 5: Cell viability in response to increasing doses of H_2_O_2_. MTT assay shows a significant reduction in cell viability with doses above 60 μM and 75 μM in HES3 (A) (IC_50_ 86 ± 11 μM) and ihFib3.2 (B) (IC_50_ 83 ± 14 μM) respectively (***p<0.0001). (C) SOD activity was lower in pluripotent stem cells when compared to mbMSC (*p<0.05, **p<0.01). No differences were found between H9, mb-iPSC, HES3 and ihFib3.2.
